# Remote Sensing Retrieval of Cloud Top Height Using Neural Networks and Data from Cloud-Aerosol Lidar with Orthogonal Polarization

**DOI:** 10.3390/s24020541

**Published:** 2024-01-15

**Authors:** Yinhe Cheng, Hongjian He, Qiangyu Xue, Jiaxuan Yang, Wei Zhong, Xinyu Zhu, Xiangyu Peng

**Affiliations:** School of Marine Technology and Geomatics, Jiangsu Ocean University, Lianyungang 222005, China; 2021220410@jou.edu.cn (H.H.); 2021220441@jou.edu.cn (Q.X.); 2022220249@jou.edu.cn (J.Y.); 2022220259@jou.edu.cn (W.Z.); 2022220263@jou.edu.cn (X.Z.); 2022220229@jou.edu.cn (X.P.)

**Keywords:** CTH, neural networks, MODIS, CALIPSO, remote sensing retrieval

## Abstract

In order to enhance the retrieval accuracy of cloud top height (CTH) from MODIS data, neural network models were employed based on Cloud-Aerosol Lidar with Orthogonal Polarization (CALIOP) data. Three types of methods were established using MODIS inputs: cloud parameters, calibrated radiance, and a combination of both. From a statistical standpoint, models with combination inputs demonstrated the best performance, followed by models with calibrated radiance inputs, while models relying solely on calibrated radiance had poorer applicability. This work found that cloud top pressure (CTP) and cloud top temperature played a crucial role in CTH retrieval from MODIS data. However, within the same type of models, there were slight differences in the retrieved results, and these differences were not dependent on the quantity of input parameters. Therefore, the model with fewer inputs using cloud parameters and calibrated radiance was recommended and employed for individual case studies. This model produced results closest to the actual cloud top structure of the typhoon and exhibited similar cloud distribution patterns when compared with the Cloud-Aerosol Lidar and Infrared Pathfinder Satellite Observation (CALIPSO) CTHs from a climatic statistical perspective. This suggests that the recommended model has good applicability and credibility in CTH retrieval from MODIS images. This work provides a method to improve accurate CTHs from MODIS data for better utilization.

## 1. Introduction

Clouds are an important component of the Earth’s radiation budget, and studying the formation, development, and evolution of clouds in the atmosphere is of great significance for simulating changes in the Earth’s radiation energy and the water vapor cycle [1]. Thus, it is essential to accurately and consistently monitor clouds during both day and night in order to capture the temporal evolution of clouds throughout the CTH. CTH is one of the important parameters of clouds for the analysis and early warning of extreme weather conditions such as thunderstorms, altitude allocation in aviation forecasts, and data assimilation in weather forecasts [2].

At present, many CTH retrieval methods from passive satellite images have been proposed and applied. The retrieval methods are usually divided into two main categories: geometric methods and physical methods. The geometric methods use a stereo-imaging technique to determine CTH [3,4,5,6,7]. For instance, MISR determines CTH with this method and the significant advantage of CTH retrieval is that this technique is not sensitive to the actual value of the observed radiances and the CTH has little bias and a standard deviation of about 1 km [8,9,10,11,12,13,14,15]. When a cloud is detected, the dominant error is from wind correction in CTH retrieval. Another method is a physical approach to determine the CTH based on the observed visible or infrared radiance, including the CO2 slicing and split window technique [16], single- or muti-channel radiance fitting [17], oxygen absorption band method [18], and optimal estimation [19,20,21]. However, these methods are applied for specific types of clouds and there are certain deviations in the CTH retrieval [2,22,23,24,25,26,27,28]. For example, Fritz et al. first proposed using the brightness temperature of the 10–12 μm infrared channel to calculate the true temperature and obtain the CTH by referring to the atmospheric temperature profile [29]. However, this method is more suitable for retrieving the CTH of thicker clouds. Some passive remote sensors, such as the Advanced Himawari Imager (AHI), provide daytime CTH information, because they only give observational data of the visible and infrared bands [29,30,31,32]. And CO2 slicing is not applicable for the retrieval of multi-layer clouds, due to their sensitivities to surface reflectance [33,34].

The Moderate-Resolution Imaging Spectroradiometer (MODIS) is one of the important passive remote sensors and provides a large range and the longest-running stable records. There are two MODIS sensors on board the Terra (EOS AM) and the Aqua (EOS PM) satellite respectively, launched by NASA. Multiple physical methods for CTH retrieval have been developed, including the CO2 slicing technique and infrared remote sensing inversion technique based on the 11 μm band IR temperature in conjunction with the National Centers for Environmental Prediction (NCEP) Global Data Assimilation System temperature profiles. However, an evaluation of MODIS CTH against collocated International Space Station Cloud Aerosol Transport System (ISSCATS) lidar observations has shown that the precision of MODIS is driven by the forward-modeling uncertainty, with about −540 ± 690 m [35]. In the previous data versions (Collection five) before 2012, MODIS retrieval CTHs showed a reported deviation from CALIPSO results of approximately −1.4 ± 2.9 km [36]. Specifically, the CO2 slicing method leads to an overall negative CTH bias, when geometrically thin high clouds exist [8]. This means that the retrieved CTHs are systematically lower than the observed results. Besides those, some studies have indicated that low-level clouds exhibit higher errors in the CTH retrieval, and a portion of the random errors is attributed to the inaccurate lapse rates [36,37]. Many previous studies have reached a consensus that the presence of multi-layer clouds can lead to significant differences in MODIS CTH retrieval, because of the limited vertical detectivity [9,11,12,13]. Therefore, it is necessary to improve the accuracy of CTH retrieval from MODIS data to provide better data for climate models and weather forecasts and so on.

Active sensors such as Cloud Profiling Radar (CPR) and CALIOP were used to acquire a comprehensive vertical profile structure of clouds and aerosols along the track based on the backscattered or reflected light, or the microwave portion of the electromagnetic spectrum [38,39,40]. These sensors are typically sensitive to cirrus clouds and can provide better cloud information [41,42]. The reliability of cloud information provided by the active sensors has been validated as a valuable resource [43,44,45]. For instance, Deng et al. conducted experiments to retrieve the composition of tropical cirrus clouds and verified cloud and climate coupling based on CloudSat and CALIPSO lidar data [44]. Wang et al. combined MODIS and CloudSat/CPR data to retrieve vertical cloud reflectivity [46]. Yang et al. also corrected the MODIS CTH of cirrus clouds in the Beijing–Tianjin–Hebei region with the CALIPSO satellite data and achieved good results [28]. Therefore, active remote sensing can provide abundant and relatively accurate information about clouds, especially the CALIPSO lidar. However, CALIPSO only measures along-track nadir points, and their spatial coverage is narrow. So, it is essential to develop a CTH identification algorithm from MODIS data to use CALIOP-like active remote sensing data. This approach complements each other’s advantages and enhances the accuracy of CTH retrieval.

In recent years, neural networks have been widely applied in many fields and have demonstrated their effectiveness as a powerful tool for extracting cloud or other meteorological parameters [47,48,49,50,51,52,53,54,55]. Training neural network algorithms often requires large amounts of good-quality data to produce accurate results, such as in modeling nonlinear problems and automating tasks. For example, Kox et al. developed an artificial neural network algorithm with SEVIRI data and CALIOP data to retrieve the CTH and cloud optical thickness of cirrus clouds [49]. Milstein et al. successfully obtained air temperature and humidity using neural networks [50]. Holl et al. trained a neural network using several datasets including CALIOP, CPR, AVHRR, and MHS to determine the ice cloud water path from AVHRR and MHS observations [51]. Stengel and Nina et al. detected the clouds and evaluated cloud parameters based on neural network models [52,53]. Minnis et al. retrieved nighttime cloud optical thickness using neural networks [54]. Loyola also employed neural networks to retrieve CTH and cloud top albedo [55]. With neural network methods, different network architectures can be built to capture the inherent physical relationship among the data itself, so following Strandgren’s advice, we selected different input parameters and built network models to improve the retrieval accuracy from MODIS data and minimize training costs [56].

CTH retrieval networks models are proposed based on the CALIOP data and MODIS data in Section 3, and Section 2 introduces the data and data processing. The remainder of this paper is arranged as follows. Section 4 gives the results of comparative analysis on the models. Finally, the discussion and conclusions are summarized in Section 5, along with proposed directions for future research.

## 2. Data and Matching

### 2.1. Aqua-MODIS Data

Terra and Aqua are two important sun-synchronous polar-orbit satellites in the US Earth Observing System (EOS) project, carrying the Moderate-Resolution Imaging Spectroradiometer (MODIS) [57]. Terra crosses the equator from north to south at 10:30 am, known as the morning star, while Aqua crosses the equator from south to north at 1:30 pm, known as the afternoon star. Terra and Aqua scan the entire Earth every one or two days and collect data from 36 spectral bands (Table 1). This study mainly uses data detected by the Aqua satellite. These observation data contain characteristic information of land, ocean, and atmospheric environments.

MODIS is mainly designed to provide large-scale global change monitoring, including global cloud cover, radiation budget changes, and dynamic processes in the ocean, land, and lower atmosphere. The radiance and reflectance data for each band are shown in Table 1, including Aqua-MODIS L1-level data products MYD02 and MYD06. Different methods are used for different atmospheric and marine parameters. The MODIS cloud data product MYD06 is especially obtained by the CO2 slicing technique including atmospheric parameters such as CTH and CTP [17]. Thus CTP is converted to height through the Global Data Assimilation System (GDAS) of the National Center for Environmental Prediction (NCEP) [58]. For ice clouds, the MODIS phase algorithm uses the 13 and 14 μm bands to retrieve cloud top pressure. For low clouds, the combination of the 11 μm band and the infrared window method, along with a latitude-related decay rate, is used to determine cloud top pressure over the ocean [58]. On land, CTP is determined by fitting the brightness temperature of band 11 with the temperature profiles provided by the GDAS [17].

The US NASA Earth Science Data System (ESDS) provides L1 and L2 observation data products of the Aqua satellite and also provides a narrow swath subset dataset of Aqua-MODIS pixels near the CALIPSO flight track, named MAC. MAC02 is the calibrated observation data, including the observation radiance of the 22 MODIS bands and MAC06 is the cloud product data, including macro- and micro-parameters related to clouds such as CTP, CTH, cloud phase infrared (CPI), cloud optical thickness (COT), cloud emissivity (CE), and so on. Table 2 shows the cloud parameters used as feature parameters for learning. MAC03 is a geographic position file that contains geographic location information. All the above datasets have a resolution of 1 km.

The MODIS instrument has two modes: daytime and nighttime. In daytime mode, all 36 bands are transmitted to the ground including both reflective solar bands and thermal emissive bands. Reflective solar bands refer to bands within the solar spectrum range that obtain information by measuring the reflection of sunlight. Thermal emissive bands refer to bands within the infrared spectrum range that obtain information by measuring the thermal radiation emitted by the Earth’s surface and atmosphere [59]. In nighttime mode, only 26 emission bands are transmitted. To ensure the comprehensiveness and comparability of all models, this study uses data from the daytime mode when creating the datasets.

### 2.2. CALIOP Data

CALIPSO was a joint NASA (USA) and CNES (France) environmental satellite, built in the Cannes Mandelieu Space Center, which was launched atop a Delta II rocket on 28 April 2006. It was equipped with three instruments, the CALIOP Lidar, the Imaging Infrared Radiometer (IIR), and the Wide Field Camera (WFC). CALIPSO is mainly used to study the impact of clouds and aerosols on Earth’s radiation budget and climate. It is the first satellite equipped with a dual-band cloud–aerosol orthogonal polarization lidar with 532 nm and 1064 nm bands and was launched in April 2006, and it provides high-quality profiles of the vertical structure of aerosols and clouds [60]. Its repetition cycle is 16 days and its overpass time is 01:30/13:30 local solar time at the equator, which is consistent with the Aqua satellite in the United States.

CALIPSO combines active lidar instruments with infrared and visible imaging instruments to generate high-vertical-resolution-profile products for detecting clouds and aerosols and studying the properties of thin clouds and atmospheric aerosols. CALIPSO satellite product data include parameters such as CTH and cloud base height. It can provide information on up to 10 cloud layers in the vertical direction.

Previous research has shown that CALIOP has a higher sensitivity to thin clouds than MODIS, and it can be used to improve the accuracy of MODIS CTH inversion [28,61,62,63]. Therefore, we use the CTHs detected by CALIOP and analyze the cloud types to train our models. The consistency between MODIS and CALIPSO data is high due to their inclusion in NASA’s A-Train satellite series, which enables us to observe the same area nearly simultaneously. The transit time difference between the two is about 80 s, which is basically consistent in time. This study mainly uses the CALIPSO satellite L2 data product from 2008 to 2009, with a horizontal resolution of 1 km and a vertical resolution of 30 m.

### 2.3. Data Preprocessing and Matching

It is relatively easy to match MODIS and CALIPSO data. In terms of space, MODIS data and CALIPSO data are matched according to the nearest-neighbor matching principle to ensure that MODIS and CALIPSO are data for the same observation location. At the same time, the error is controlled within 3 min in time to complete the data matching. MODIS uses a scanning radiometer measurement method and has global coverage capabilities. CALIPSO uses a nadir-pointing lidar detection imaging method and can only cover a narrow band area below the orbit. Therefore, not all MODIS pixel points can be matched one-to-one with CALIPSO data. In this study, pixel point matching with CALIPSO is found in the MODIS dataset along the nadir point of the CALIPSO orbit.

**Figure 1:**
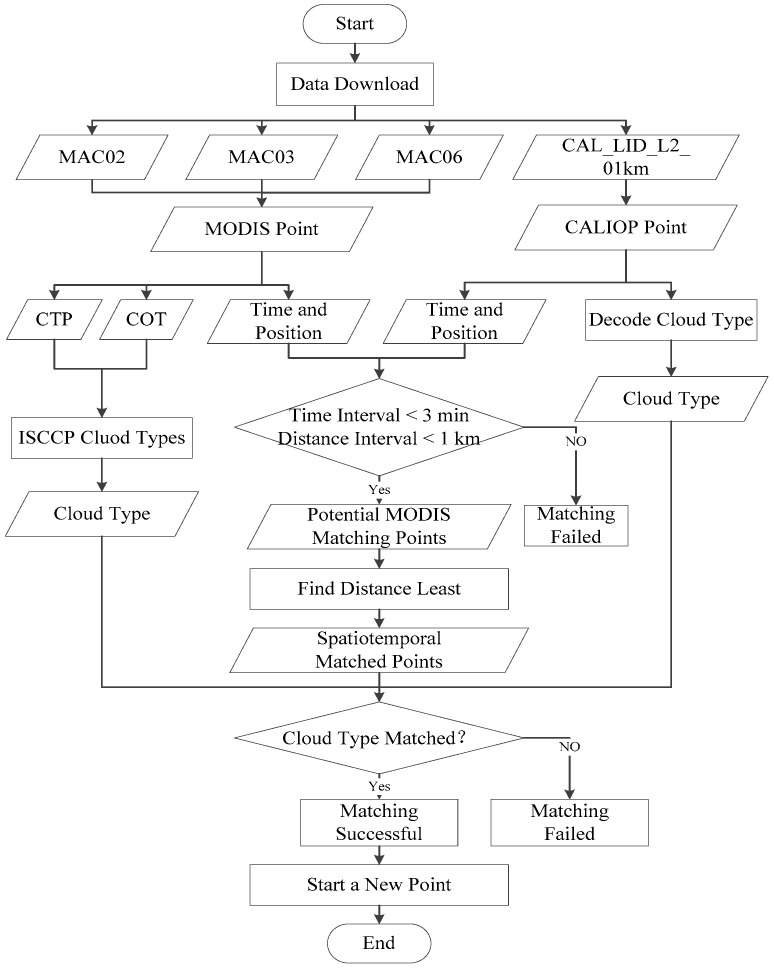
Matching process between MODIS data and CALIOP data.

Before data matching, geographic data fusion is performed on MAC02, MAC06, and MAC03 to obtain MODIS points, as shown in Figure 1. Since there is no location information in MAC02 and MAC06, matching with CALIPSO data is impossible. It needs to be read from MAC03 to form standard data with a resolution of 1 km carrying location information. Then, it uses the time and position from MODIS and CALIOP data to control time and distance intervals. In many potential matching points, the least distance point is matched.

For spatiotemporal matched points, it needs to perform a cloud type check, which means that the same type of cloud point is adopted. Due to differences in sensor detection principles, there may be inaccuracies in the detection information. Therefore, cloud type matching is necessary to ensure data accuracy, meaning that the chosen points are consistent with the observation results of MODIS and CALIPSO. Forsythe et al. proposed that clouds of the same type have the same or similar cloud heights and preliminarily verified the superiority of cloud height estimation schemes based on this assumption using satellite cloud type products and ground observation station cloud height data [64]. Wang and Yu et al. further verified the feasibility of estimating cloud height based on the same cloud type on this basis [65,66]. Therefore, after nearest-neighbor spatiotemporal matching, this study performs the same cloud type matching between MODIS pixel points and CALIPSO data pixel points.

**Figure 2:**
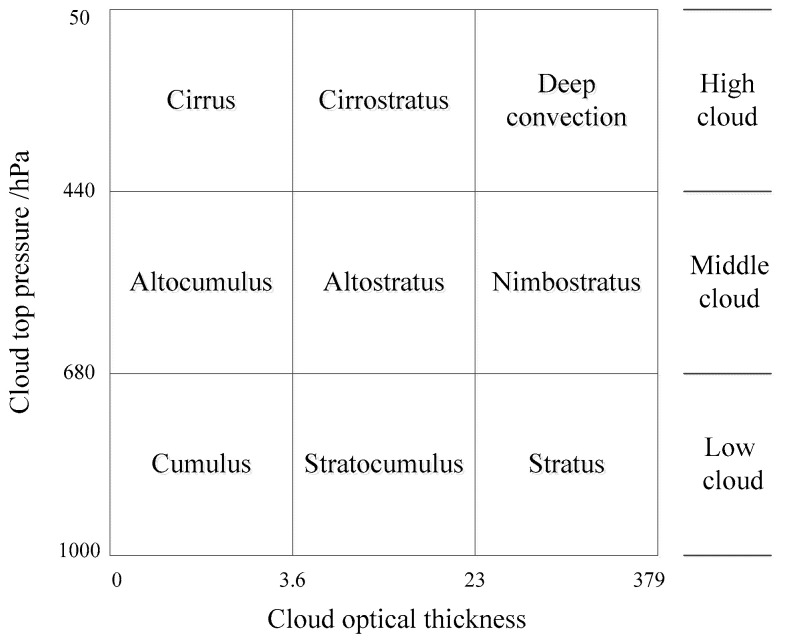
Cloud genera from MODIS data according to the ISCCP cloud classification criteria.

Since MODIS does not provide cloud type products, the cloud classification scheme of the International Satellite Cloud Climatology Project (ISCCP) is adopted to classify MODIS pixels. ISCCP classifies clouds into nine categories based on CTP and cloud optical thickness, with the remaining categories marked as cloudless, as shown in Figure 2 [67]. Thus, MODIS data related to cloud parameters are grouped into three classes, including low cloud, middle cloud, and high cloud. Similarly, according to the cloud information provided by the Data Processing Center (DPC), CALIPSO clouds are interpreted and classified into different cloud types as Figure 2 shows [68]. CALIPSO data are recorded into 8 classes, and according to its cloud features of each class, they are grouped into three categories, low cloud, middle cloud, and high cloud, as shown in Table 3.

For the same cloud genera, MODIS data are matched with the corresponding CALIPSO CTH. When CALIPSO provides more than one cloud type, the highest CTH is chosen. For example, the rain layer clouds and high clouds from MODIS data are matched with the opaque high clouds identified by CALIPSO, and they are recorded as high clouds. The cirrus and cirrostratus clouds from MODIS are matched with the transparent cirrus clouds from CALIPSO, and they are labeled as low clouds. Other cases are marked as cloudless.

After geographic data fusion and nearest-neighbor spatiotemporal and cloud type matching, four datasets are generated. Among them, the 2008 data are used to generate training and testing datasets. According to general machine learning experience, this study randomly generates training and testing datasets in an 8:2 ratio to ensure that the data distribution of the two datasets is consistent. The full-year data of 2009 are used as a validation dataset to validate the model. In addition, a case dataset is created from one MODIS image at 4:45 Coordinated Universal Time (UTC) on 9 August 2019 to examine the generalization ability of the models. The specific information of the dataset is shown in Table 4.

**Figure 3:**
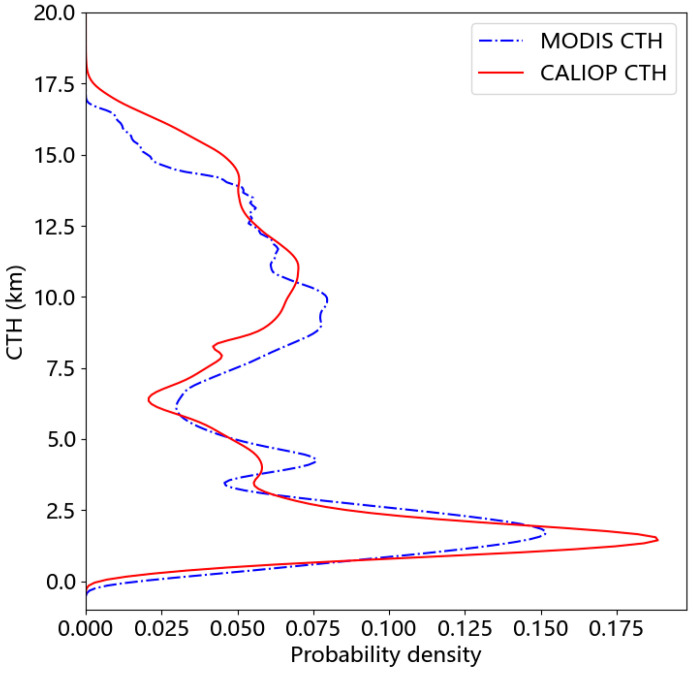
Data distribution of CTHs for MODIS and CALIOP in 2008.

Figure 3 shows the data distribution frequency of CTHs from MODIS and CALIOP in 2008 and they are very close. This indicates that the data distribution is reasonable and the data are reliable.

## 3. Algorithms and Models

### 3.1. Correlation Analysis of Feature Parameters

Before establishing the model, the correlation between each variable and CTH is analyzed using correlation analysis tools. This study uses the Pearson Correlation Coefficient (PCC) to measure the correlation between variables, and its calculation is as follows:

(1) $$PCCs=\frac{\sum x_{i}y_{i}-n\bar{x}\bar{y}}{{(n-1)S}_{x}S_{y}}=\frac{n\sum x_{i}y_{i}-\sum x_{i}\sum y_{i}}{\sqrt{n\sum x_{i}^{2}-{\left(\sum x_{i}\right)}^{2}\;}\;\sqrt{n\sum y_{i}^{2}-{\left(\sum y_{i}\right)}^{2}}}$$

**Figure 4:**
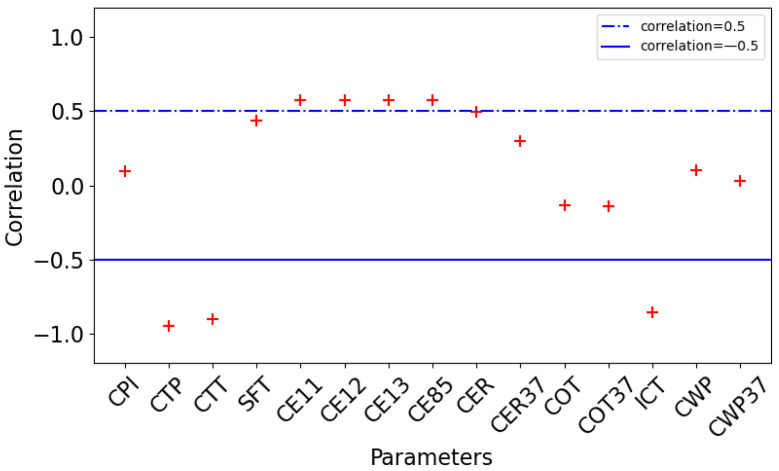
Correlation between MODIS L2 data cloud parameters and CALIOP CTH.

The PCC checks the direction and degree of the change trend between variable x and variable y. The value is within the range of [–1,1]. 0 indicates that there is no correlation between the two variables being checked. A positive value indicates a positive correlation and a negative value indicates a negative correlation. The larger the value, the stronger the correlation. Figure 4 shows the correlation between each MODIS product parameter and CTH.

As shown in Figure 4, the correlation coefficients of CTP, CTT, and ICT are close to −1, which indicates that there is a strong negative correlation between CTP, CTT, ICT, and CTH. In addition, SFT, CE11, CE12, CE13, CE85, and CER have a positive correlation around 0.5. The correlation coefficients of CPI, cloud optical thickness (COT, COT37), and cloud water path (CWP, CWP37) are low, that is, the linear correlation between each parameter and CTH is very weak. However, considering the actual situation, cloud phase and other factors may also reflect the situation of CTH and can be considered for learning.

**Figure 5:**
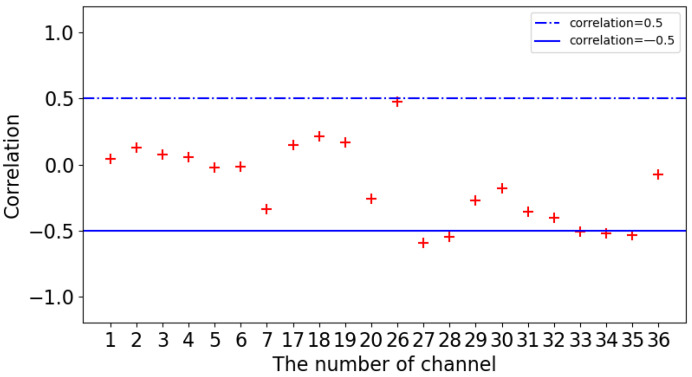
Correlation between MODIS L1 radiance and CALIOP CTH.

Figure 5 shows the correlation between the radiance of each band and CTH. Overall, the correlation coefficient of L1 radiance parameters is lower than that of L2 cloud parameters, indicating that L2 cloud parameters are more suitable for the inversion of CTH. From the above analysis, the correlation coefficients of bands 1–7 are close to 0 and have no linear relationship with CTH. The correlation coefficients of bands 27–28 and 33–35 are relatively high and have a relatively strong correlation with CTH. When establishing the model, feature parameters are selected according to this analysis. Selecting parameters with a higher correlation and giving them greater weight are conducive to the learning and convergence of the model.

### 3.2. Building and Training of Models

Since machine learning can model complex nonlinear problems and has extremely strong feature extraction capabilities, it has been widely used in the retrieval of cloud parameters. Classic machine learning models include support vector machines (SVMs), backpropagation neural networks (BP), convolutional neural networks (CNNs), and recurrent neural networks (RNNs). Different models are suitable for dealing with different problems. Previous studies show that BP neural networks exhibit commendable performance in the retrieval of CTH [69,70,71]. Therefore, we choose the BP neural networks to build the models.

**Figure 6:**
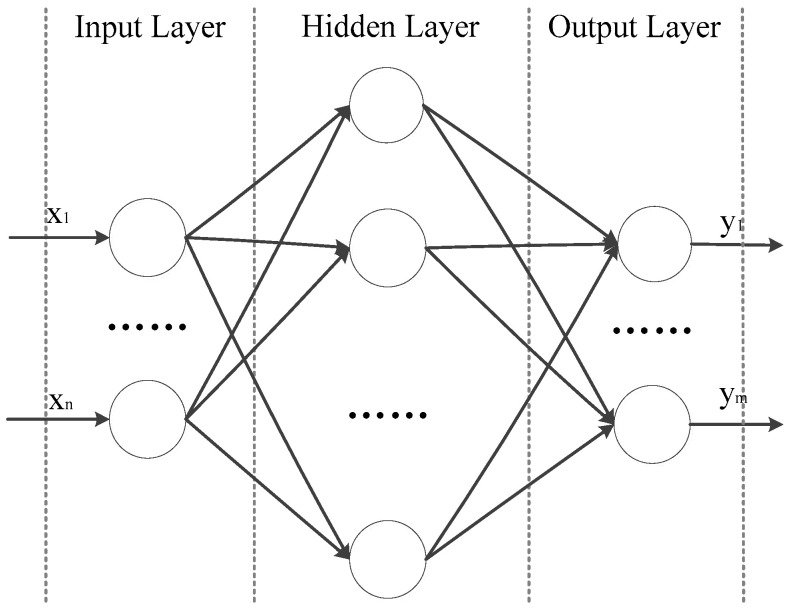
Architecture of backpropagation neural networks.

The architecture of a feedforward neural network consists of three types of layers: the input layer, hidden layers, and the output layer. Each layer is made up of neurons, and the layers are interconnected by weights. Each neuron has an independent weight for controlling information transmission and transformation. The weights are trained through the backpropagation algorithm, allowing the network to learn the mapping relationship between the input and output and achieve a nonlinear function approximation. As shown in Figure 6, the input layer is composed of *n* neurons and is represented by $x_{i}(i=1,2,\ldots ,n)$, which is also the output layer. The hidden layer has q neurons and is represented by $z_{k}(k=1,2,\ldots ,q)$ as the output of the hidden layer. The output layer is composed of m neurons and is represented by $y_{j}(j=1,2,\ldots ,m)$ as the output of the output layer.

There are three types of inversion algorithms proposed in this paper. The first type uses the MODIS L2 cloud parameter product data as input, including various cloud parameters such as cloud top pressure, cloud top temperature, cloud optical thickness, and cloud emissivity. The second type employs the MODIS L1 calibrated radiance product data, which include calibrated radiance data from multiple visible and infrared bands. The third type combines selected cloud parameters with calibrated radiance data. All three types of models select a total of seven sets of feature parameters as inputs to the neural network model, with CTH as the output. The specific parameters selected are shown in Table 5. CALIPSO’s CTH is used as the ground truth to develop the models, which have four hidden layers with 128, 360, 360, and 128 nodes. The Adam optimizer is used during training with an initial learning rate of 0.0001.

According to Table 5, model 1 is a full parameter model that uses 12 cloud parameters as inputs, including cloud top pressure, temperature, and so on. Parameters such as CER, COT, and CWP are calculated using reflective solar bands and are unavailable in nighttime mode. Model 2 uses only 8 radiation band parameters to achieve universality in different modes. Model 3 uses 8 cloud parameters.

**Figure 7:**
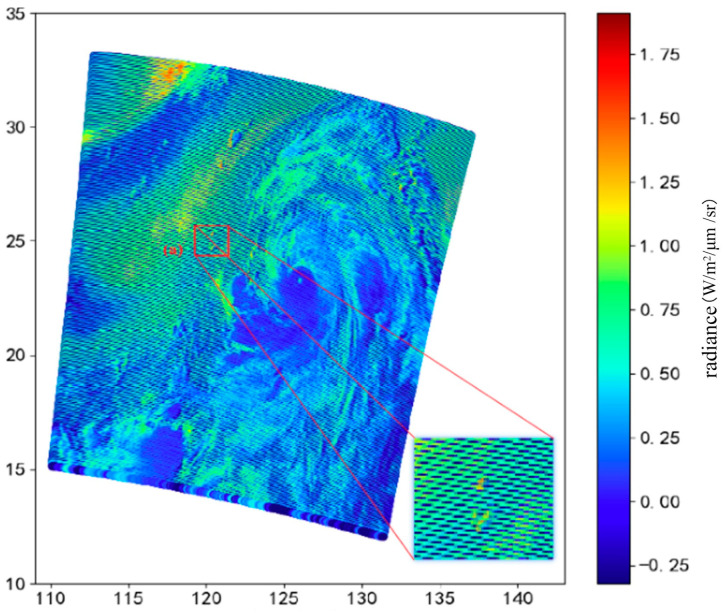
Radiance of 32nd band from the Terra satellite at 2:30 UTC on 8 August 2019. The clear noise pixels are shown in the enlarged view. The colors represent the magnitude of the radiance values.

In addition to the contribution of cloud parameter information to CTH, this paper also considers the inversion of CTH directly through the original radiance values, so model 4 and model 5 are designed. MODIS radiance values are provided by the L1-level product, of which 22 bands are related to cloud parameters and the 30–36th bands are designed to observe CTH information. Models 6 and 7 both use band radiance and their cloud product parameters. It is worth noting that in the MODIS image, some bands have data with “stripe noise” or “grain noise”. During the detector’s sampling process, some detectors may have high or low response values, resulting in obvious noise pixels in the image. For instance, Figure 7 displays 32nd band data from the Terra satellite captured on 8 August 2019, which clearly exhibits noticeable noise in the L1-level MODIS data.

During the model training process, features like noise pixels may be removed. Shen et al. proposed an improved Bayesian learning algorithm based on compressed sensing and tested it on simulated Terra and real Aqua images with good results [72]. To avoid affecting inversion results, the model can be designed to avoid noise bands and low-correlation bands. Model 4 includes all band radiance values, while model 5 avoids selecting noisy bands and low-correlation bands. Our analysis of cloud parameter correlations reveals a strong correlation between cloud top temperature, pressure, and height. This paper considers combining these two parameters with MODIS-calibrated radiance data to train a more accurate CTH inversion model and designs the third type of model. Therefore, based on model 5, model 6 adds two cloud parameters: CTP and CTT. Similarly, MODIS bands are reflective solar bands and thermal emissive bands. In nighttime mode, information on reflective solar bands cannot be obtained. Therefore, the feature parameters of model 7 are selected to ensure the universality of the model.

(2) $$z=\frac{x-\mu }{\sigma }$$

The feature parameters in Table 5 are input into the models. Before training, the dataset is standardized to scale the data to a distribution with zero mean and unit variance, eliminating the difference in dimensions between different features and allowing the model to better learn the patterns in the data. As shown in Equation (2), x represents the original data, μ represents the mean of the data, σ represents the standard deviation of the data, and z represents the standardized data. Each sample point can have an impact on standardization. Subsequently, the standardized data are input into the model for training.

### 3.3. Evaluation Metrics of Models

The performance of the models is evaluated with the test dataset. The main evaluation metrics used are the Mean Absolute Error (MAE), Mean Squared Error (MSE), Root-Mean-Squared Error (RMSE), and goodness of fit ($R^{2}$). These metrics are used to measure how well the model predictions match the actual values, with the MAE, MSE, and RMSE indicating the error between predicted and actual values, and $R^{2}$ indicating how well the model fits the data. Particularly, *MAE* measures the absolute value of the difference between the predicted value and the true value. The calculation formula is shown in Equation (3). ${\hat{y}}_{i}$ denotes the models’ predicted CTHs and $y_{i}$ is the observed value from CALIOP.

(3) $$MAE=\frac{1}{m}\sum_{i=1}^{m}\left|y_{i}-{\hat{y}}_{i}\right|=\frac{1}{m}\sum_{i=1}^{m}\left|e_{i}\right|$$

*MSE* reflects the expected value of the difference between the predicted value and the true value, it can measure the average error well, and it is used to evaluate the degree of data change. The smaller the *MSE* value, the higher the prediction accuracy of the model and the better the generalization effect of the model. *RMSE* is the square root of *MSE*. The calculation formulas are shown in Equations (4) and (5), respectively.

(4) $$MSE=\frac{1}{m}\sum_{i=1}^{m}{\left(y_{i}-{\hat{y}}_{i}\right)}^{2}$$

(5) $$RMSE=\sqrt{MSE}=\sqrt{\frac{1}{m}\sum_{i=1}^{m}{\left(y_{i}-{\hat{y}}_{i}\right)}^{2}}$$

$R^{2}$ means goodness of fit and represents the degree to which the model explains the variation in the dependent variable and reflects the fitting effect of the predicted and true values. The higher the $R^{2}$, the better the fitting effect. The formula for calculating $R^{2}$ is as follows:

(6) $$R^{2}\left(y_{i},{\hat{y}}_{i}\right)=1-\frac{\sum_{i=1}^{m}{\left(y_{i}-{\hat{y}}_{i}\right)}^{2}}{\sum_{i=1}^{m}{\left(y_{i}-{\bar{y}}_{i}\right)}^{2}}$$

The above indicators are used to comprehensively evaluate the models and determine the optimal model for CTH retrieval. This model is then applied to analyze and perform CTH retrieval.

## 4. Results

### 4.1. Statistic Analysis of Retrieval Performance

**Figure 8:**
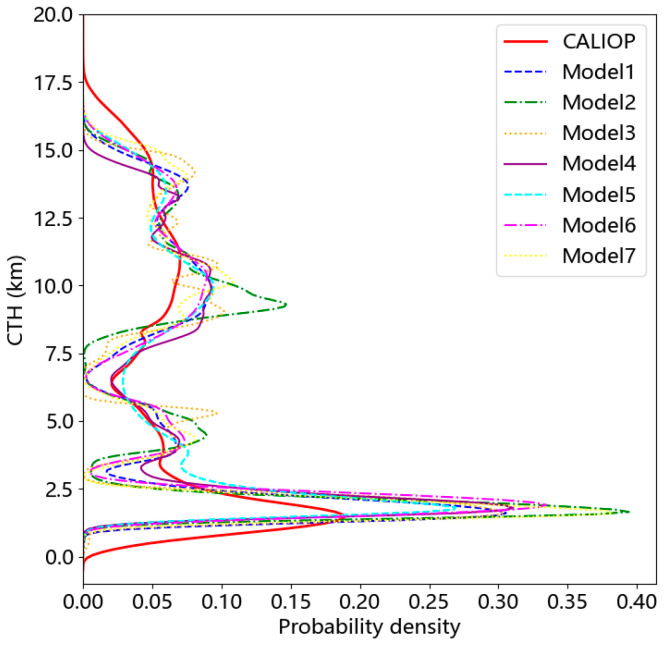
Data distribution of CTHs for CALIOP and models 1–7 in 2009.

MODIS mainly detects radiation and reflection, while CALIOP can provide the vertical structure information of clouds. Therefore, the CTHs from CALIOP are more accurate, and they were used as the observed values. In this study, three types of different algorithms were designed and a total of seven models were trained. The results were calculated from the year-round data of 2009. The data distribution of CTH for CALIOP and models 1–7 is shown in Figure 8. The distribution curves of all model results evidently show a substantial discrepancy with CALIOP when it comes to low clouds at about 0–3 km. This implies that the precision of the models’ inversion for low clouds leaves room for improvement.

**Figure 9:**
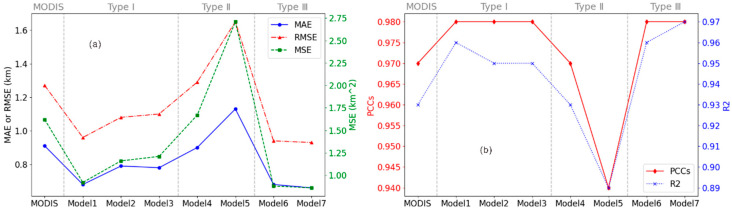
Evaluation of parameter performance of the errors including MAE, RMSE, and MSE (**a**), and the goodness of fit ($R^{2}$) and correlation (PCC) (**b**).

The MAE, MSE, RMSE, PCC, and $R^{2}$ between the model results and the CALIPSO CTHs were calculated and compared, as shown in Figure 9.

As Figure 9a shows, the errors of the models of types I and III are lower than that of the MODIS CTH product, which indicates that the models of types I and III effectively improved the accuracy of CTH retrieval. The models of type II have larger errors compared with the other models and MODIS product, which indicates that model 4 and 5 of type II do not have an advantage. Similarly, from the goodness of fit and correlation, the larger the magnitude of those two parameters, the better the performance of the models. From Figure 9b, all models boast $R^{2}$ values exceeding 0.89 and PCC values surpassing 0.94. This indicates that all models have undergone comprehensive training, achieving a high degree of data fitting and capturing the linear relationships between variables. They demonstrate exceptional data fitting capabilities, exhibiting a high level of consistency in both $R^{2}$ and the PCC. However, type II models present lower $R^{2}$ and PCC values compared to the models of types I and III. This suggests that models 4 and 5 may underperform relative to the others.

In fact, the models of types I and III boast a greater $R^{2}$ and PCC, and they outperform MODIS CTHs in terms of $R^{2}$ and the PCC, attesting to the reliability and proficiency of these models in CTH retrieval from MODIS data. Conversely, the R^2^ and PCC of type II, with minima of 0.89 and 0.94, respectively, are also larger than those of the others, which validates the above conclusion. Additionally, a *PIrmse* parameter was utilized to quantify the extent of model improvement, with the following formula:

(7) $$PIrmse=\frac{{RMSE}_{MODIS}-{RMSE}_{Model}}{{RMSE}_{MODIS}}\times 100\%$$

In Table 6, model 6 and model 7 of type III reduced the errors by 26.4% and 27.3%, respectively, compared to the MODIS product, followed by the models of type I. This indicated that the method of combining calibrated radiance data and cloud parameters to retrieve CTH had good performance and higher accuracy. However, in contrast to type III, type II without the CTP and CTT inputs yielded worse results, with the average RMSE increasing by 17.9%. This shows that CTP and CTT are key parameters and play an important role in the retrieval of CTH from MODIS data.

**Figure 10:**
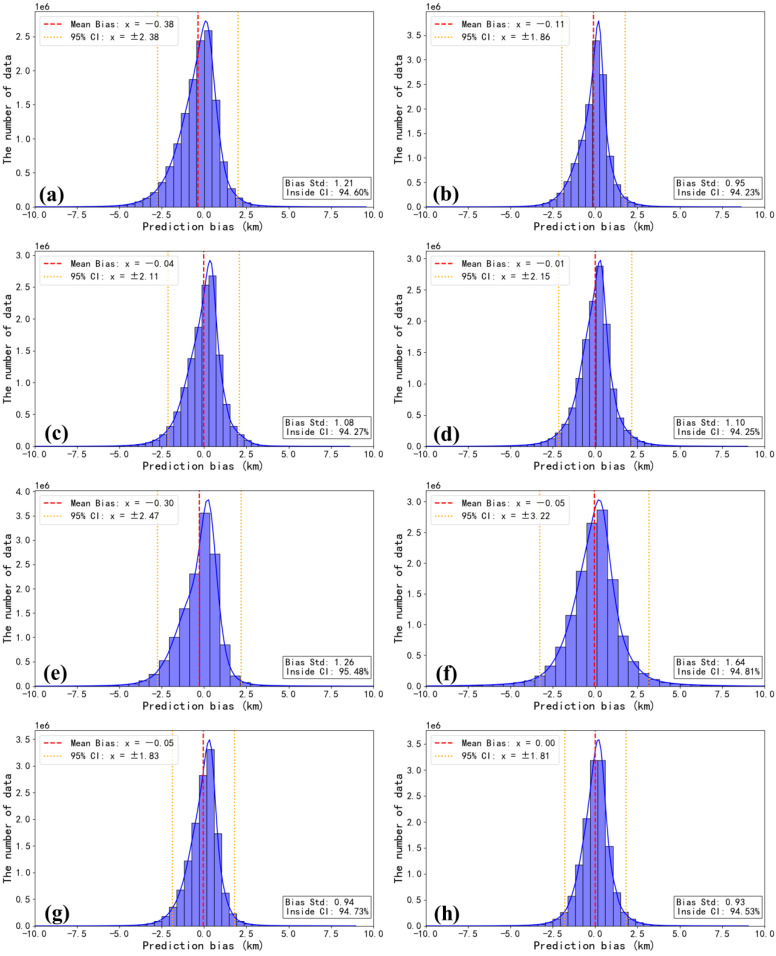
Distribution of the differences between CALIOP CTHs and those from MODIS (**a**), model 1 (**b**), model 2 (**c**), model 3 (**d**), model 4 (**e**), model 5 (**f**), model 6 (**g**), model 7 (**h**).

In addition to the model error, goodness of fit, and correlation, it is essential to consider the distribution of bias. The differences between CALIOP CTHs and other results were calculated as the prediction bias and the 95% confidence intervals are plotted in Figure 10. The narrower the 95% confidence interval of the sample, the better the accuracy of the models. A narrower confidence interval indicates smaller random errors, resulting in a more precise outcome.

For all models, more than 94% of the results fall within these confidence intervals, which shows further confirmation of the adequacy and reliability of the model training. However, they differ in confident interval and prediction bias. Notably, the confidence intervals for the models of type I and type III are narrower than that of the MODIS product, effectively reducing the uncertainty in CTH retrieval. Conversely, from Figure 10e,f, the confidence intervals of type II are wider than those of the others, which again validates the above conclusion. Of course, among all the models, model 7 is the best with the 95% narrowest confidence interval and near-zero bias.

**Figure 11:**
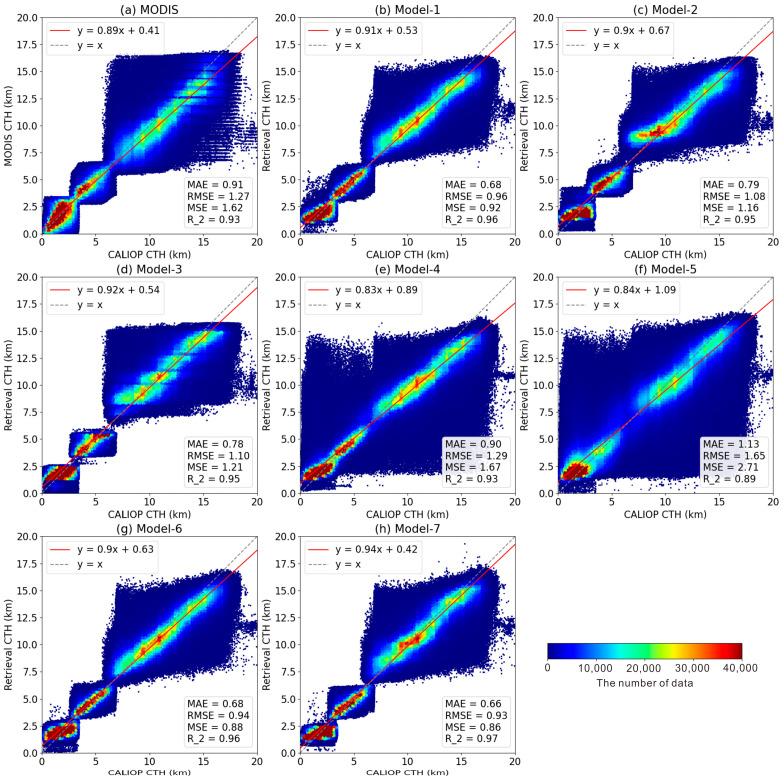
Scatter plot of CALIOP CTHs and those provided by MODIS (**a**), model 1 (**b**), model 2 (**c**), model 3 (**d**), model 4 (**e**), model 5 (**f**), model 6 (**g**), and model 7 (**h**).

Finally, as depicted in Figure 11, a set of scatter plots was made to analyze the applicability of the model. In Figure 11, the red line is the fitting line of the scatter plot and the gray dashed line is y = x. The smaller the angle between the two lines, the closer $R^{2}$ is to 1 and the more accurate the retrieval results are. It is easy to observe that the degree of dispersion of the scatter plot is closely related to the size of the errors, including the MAE, MSE, and RMSE. Usually, a lower error value means that the scatter is more concentrated near the y = x line, as shown in Figure 11b–d,g–h. This once again demonstrates the effectiveness of the models of types I and III.

It is worth noting that the scatter points of type II (models 4, 5) are relatively dispersed with larger errors, which indicates that it is very difficult to obtain an excellent retrieval result based solely on calibrated radiance. Based on the slope, both types of models have optimal fitting slopes exceeding 0.9, with a maximum of 0.94 for model 7 and a minimum of 0.9 for model 2 and model 6. This also indicates that model 7 of type III is the best model, followed by model 3 and model 1. This conclusion is further supported by parameters such as the average error and correlation coefficient.

Some interesting scatters are found in all rightmost subplots. The CTHs of this small portion of points show significant differences compared to the observed values, as shown in Figure 11a. CALIOP detects CTH for these points at approximately above 17.5 km, while MODIS estimates CTHs to be around 8–11 km. One possible reason for this discrepancy is the presence of thin cirrus clouds, which typically occur in overlapping cloud conditions [73]. The CALIOP is highly sensitive to capturing thin cirrus clouds located at higher altitudes, while MODIS detects comprehensive cloud information for the pixel and its surroundings, leading to the determination of a lower CTH value for this pixel. Undeniably, there are objective differences between the two datasets, but this small portion of data is of negligible quantity and therefore does not impact the overall performance of the models.

### 4.2. Statistical Analysis of Retrieval in Different Cloud Types

It was found that the retrieved results of model 4 and model 5 were not satisfactory, while the performance of the others was not poor and model 7 is recommended. In order to evaluate the performance of the remainder in more detail, this paper discusses the retrieval accuracy of the models for different cloud genera. According to the ISCCP international cloud classification scheme, clouds have nine categories belonging to three different cloud genera, including low, middle, and high clouds, as shown in Figure 2. This paper analyzes the retrieved results of different models for each cloud genera. The following figure shows the Gaussian probability kernel density estimation curves.

**Figure 12:**
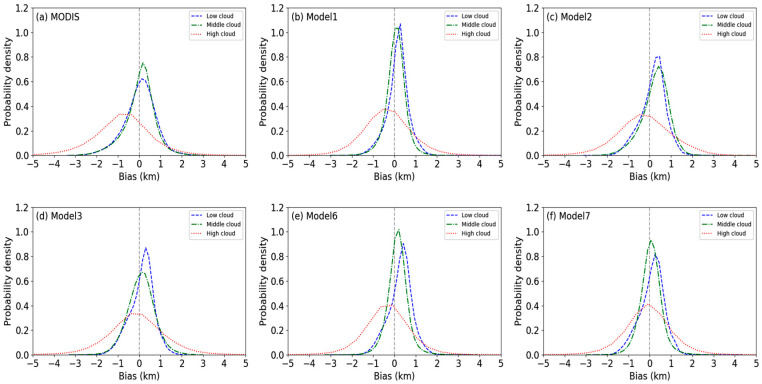
Gaussian kernel density estimation curves of prediction bias. The bias between CALIOP CTHs and other results provided by MODIS (**a**), model 1 (**b**), model 2 (**c**), model 3 (**d**), model 6 (**e**), model 7 (**f**).

Figure 12a clearly illustrates that the MODIS CTH product exhibits a significant bias for high clouds, with an overall bias ranging between −4 and 2 km. This bias predominantly manifests as a negative deviation of approximately 1 km. The peaks for low and middle clouds, which have a smaller bias, are situated near a bias of 0 km. Type I models show some improvements for high clouds. As can be observed from Figure 12b–d, the peak of the probability density curve shifts to the right, moving closer to a bias of 0 km and displaying a negative bias of less than 1 km. However, some models, such as model 2, also exhibit an increased bias for low and middle clouds, where the bias is primarily distributed around 0.5 km. Type III models demonstrate a substantial enhancement in high and middle clouds, especially model 7, where the peaks for high and middle clouds are near a bias of 0 km, with a minor discrepancy from the CALIOP true data. However, the bias of all models for low clouds increases. Considering the bias, we suggest utilizing model 7 for the retrieval of middle and high clouds and employing model 1 for the retrieval of low clouds.

**Figure 13:**
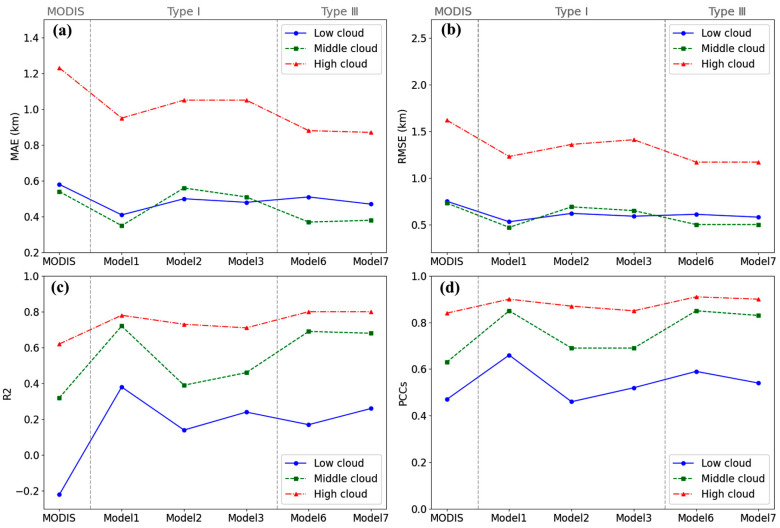
Performance with evaluation parameters including MAE (**a**), RMSE (**b**), $R^{2}$(**c**), and PCC (**d**) in different cloud genera.

However, we cannot evaluate the suitability of a model for different cloud layers solely from the perspective of bias, as the bias is influenced by the range of the data itself. For example, the bias of low clouds is always less than that of high clouds, but this does not necessarily mean that the accuracy of low-cloud inversion surpasses that of high clouds. This phenomenon may be due to the larger range of values for high clouds. Therefore, we need to refer to multiple indicators for a comprehensive evaluation of the models and calculate some parameters such as the MAE, RMSE, $R^{2}$, and PCC, as shown in Figure 13.

From the three kinds of errors, a pattern similar to bias emerges, as shown in Figure 13a–c. On the whole, the error associated with type III is lower than that of type I. Moreover, in most instances, the error of high clouds consistently surpasses those of low and middle clouds. The magnitude of errors between different cloud genera does not necessarily indicate the quality of the model’s performance, since the distribution range of sample data points differs. For example, the distribution range of low cloud data is relatively small, so there is a small error compared to those of the middle and high cloud genera. Therefore, the goodness of fit and PCC were similarly used to validate the models.

The situation differs when considering $R^{2}$ and the PCC. As depicted in Figure 13d, the $R^{2}$ and PCC for low clouds in each model are significantly lower than those for middle and high clouds, which shows that the models perform poorly in low cloud retrieval. Model 1 performs relatively well on middle and low cloud genera, with a higher $R^{2}$ and PCC. Model 6 has the best retrieval effect on high clouds with an $R^{2}$ and PCC as high as 0.80 and 0.91, respectively. Model 7 is close to model 6 in performance, with little difference in high and middle clouds, but according to PCCs, it performs slightly worse than models 1 and 6 in terms of low clouds.

Considering the overall parameters, the retrieval accuracy of each model is higher for high clouds, followed by middle clouds, and it is the worst for low clouds. Among them, models 1, 6, and 7 all perform well on high and middle clouds. For low-level clouds, model 1 proves to be the best performing model among all, exhibiting a PCC of 0.66.

### 4.3. Geographical Application of the Models

#### 4.3.1. A Case Retrieval during the Typhoon Lekima Period

From a statistical perspective, except for models 4 and 5, the remaining models demonstrate good overall performance; however, significant differences are observed in the details, such as the retrieval of low and mid-level CTHs. To validate the applicability of the models, the models were used to retrieve CTHs during the Typhoon Lekima period in 2019. Typhoon Lekima formed on the sea surface east of Luzon Island in the Philippines on 29 July 2019. It was officially named at 15:00 on 4 August and upgraded to a super typhoon at 23:00 on 7 August. It continued to move northwestward and approached the coast of Zhejiang. It landed in Zhejiang Province on the night of 10 August. At the time of landing, the maximum wind force near the center was level 16. Finally, it moved into the Bohai Sea and continued to weaken, dissipating on 13 August.

**Figure 14:**
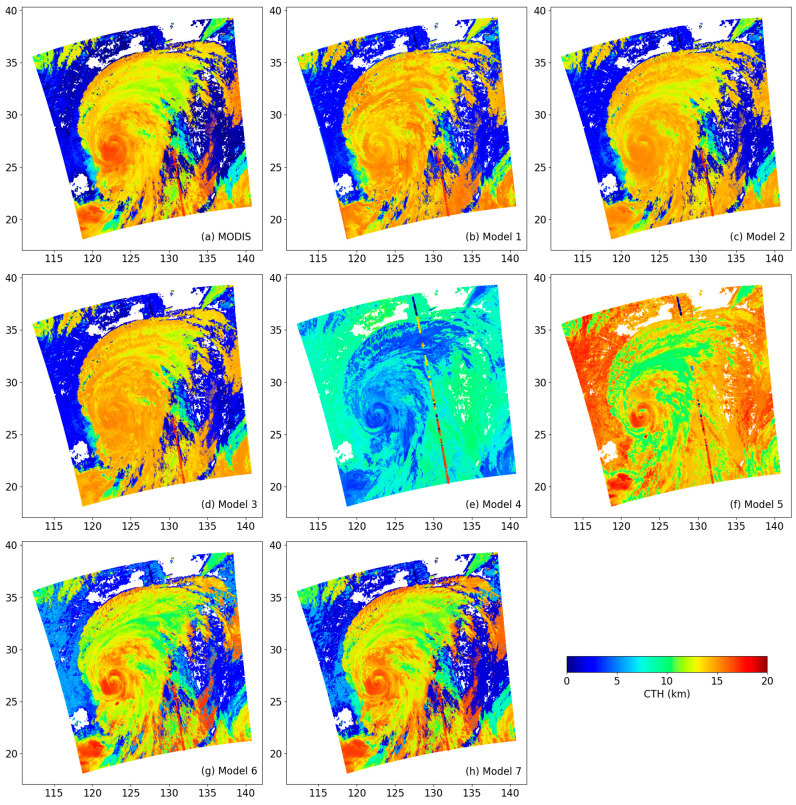
Different CTHs at 4:45 UTC on 9 August 2019 from MODIS product (**a**), model 1 (**b**), model 2 (**c**), model 3 (**d**), model 4 (**e**), model 5 (**f**), model 6 (**g**), and model 7 (**h**). The colored diagonal line represents nadir-pointing CALIPSO CTHs.

During the typhoon period, the structure of the typhoon is clearly visible from satellite cloud imagery, including high, mid-level, and low clouds. The cloud information is quite rich; thus, the MODIS image of Typhoon Lekima at 4:45 UTC on 9 August 2019 was used to retrieve CTHs using the models. Figure 14 shows the MODIS CTHs and the models’ results. Meanwhile, we provided the CALIPSO CTHs along the nadir track for the same time in Figure 14. Comparing MODIS data in Figure 14a with CALISPO data, it can be seen that the colors of the two datasets are relatively close, but in the higher cloud part in the south, CALIPSO obviously measures a higher CTH.

Figure 14 shows the different structures of Typhoon Lekima; however, the typhoon structure depicted in Figure 14e,f is clearly inconsistent with the observation. A fully developed typhoon is a deep low-pressure system and typically consists of an eye, eyewall, cloud wall, and spiral arm region. If clouds exist in the eye of the typhoon, the CTHs are lowest because the airflow is primarily descending. In the eyewall region, the CTHs are typically highest because the airflow is mainly ascending, with CTHs reaching above 12 km. The CTHs in the cloud wall and spiral arm regions are generally lower because the airflow in these areas is predominantly horizontal. Therefore, the structure of Typhoon Lekima is clearly described in Figure 14a,g,h.

**Figure 15:**
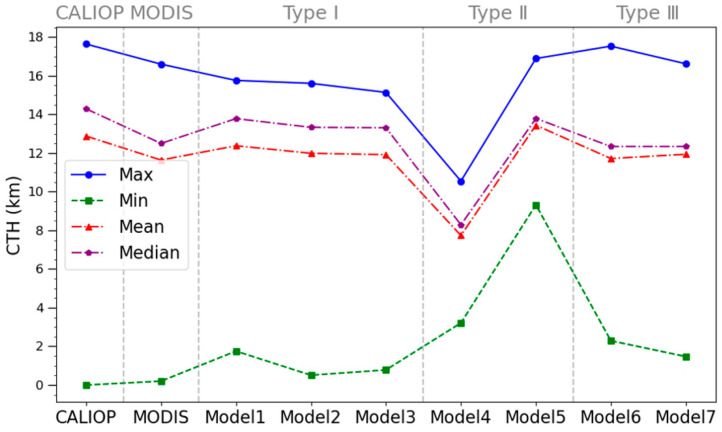
Comparison of the model CTHs with the CTHs from the CALIPSO.

However, the CTHs in the whole typhoon region are either lower in Figure 14e or higher in Figure 14f than those in the surrounding areas, so this conclusion is obviously incorrect. These results indicate that model 4 and model 5 are not suitable to retrieve the CTHs from MODIS data. In other words, the second type of model is not reasonable. As shown in Figure 14b–d, the structure of the typhoon is presented, but they show relatively uniform CTHs in space. They do not effectively distinguish between the eye, eyewall, and spiral arm regions. A previous study found that the CTHs on the south side of Typhoon Lekima in the spiral arm regions were higher than those on the north side [74]. Those results show that model 1, model 2, and model 3 are inapplicable for the retrieval of CTHs. So, we should not use the first type of models to retrieve CTH from MODIS. The remaining model 6 and model 7 are evidently quite good as they can effectively identify the different structures of the typhoon. A quantitative comparison between these models was conducted and the CTHs from the colored diagonal line in Figure 14 were calculated and are plotted in Figure 15. 

From Figure 15, regardless of the minimum, maximum, median, and mean CTH, the CTHs retrieved by model 7 are closest to those from CALIOP, followed by model 6. From Figure 14 and Figure 15, it is clear that model 7 is recommended for CTH retrieval from the MODIS among the seven models.

#### 4.3.2. Application of the Global CTH Retrieval

Another application of the model involves global CTH retrieval. Model 7 with the lowest RMSE was employed to retrieve global CTHs from the MODIS data and the CTHs were then compared with the observed CTHs from CALIPSO.

**Figure 16:**
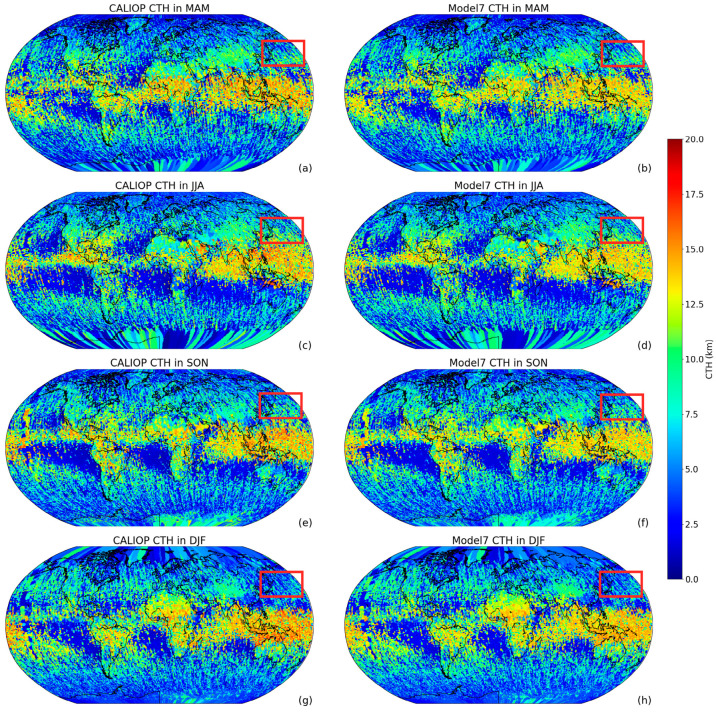
Global seasonal distribution of CTH in 2009. MAM stands for March, April, and May. Similarly, JJA represents June, July, and August; SON corresponds to September, October, and November; DJF signifies December, January, and February. The right column including figures (**a**), (**c**), (**e**), and (**g**) shows the observed CTH from CALIPSO. The left column including figures (**b**), (**d**), (**f**), and (**h**) shows the retrieved CTH from model 7. The red box in the figure indicates a part of the Northwest Pacific region.

From the spatial distribution pattern, the CTHs from the two kinds of data samples decrease from the equator towards the North and South Poles. As shown in Figure 16, the clouds are highest in low-latitude regions, followed by mid-latitude regions, and they are generally lower in high-latitude regions. In tropical areas near the equator, due to the large solar zenith angle, the surface receives a higher intensity of solar radiation. This causes the surface temperature to rise, strengthens the upward movement of air, and forms a thermal circulation within the troposphere. This upward movement causes water vapor in the air to rise and condense into clouds after cooling. In tropical areas, due to the obvious upward movement, CTHs are relatively high and common cloud types include cumulonimbus and high-level clouds. Mid-latitude regions are mainly affected by a temperate monsoon climate and continental climate. The solar radiation intensity and surface temperature in these areas are relatively low, so the upward movement of air is not as obvious as in tropical areas, and CTHs are relatively low. In high-latitude areas, due to low surface temperatures and a low water vapor content, it is difficult for water vapor in the air to condense into clouds and CTHs are generally low. The CTHs from model 7 are generally consistent with the CALIPSO values that demonstrate the feasibility of our proposed model.

The year is divided into four seasons. MAM, JJA, SON, and DJF represent spring, summer, autumn, and winter, respectively. From right to left in Figure 16, global CTH seasonal variations can be observed from the images on both sides, and the patterns of the two datasets are also consistent. In all seasons, the CTHs in the tropical zone remain nearly constant and those of the other zones exhibit noticeable seasonal variations. For example, the Northwest Pacific region was chosen (the red box in Figure 16) and the average CTHs were calculated from two datasets in 2009 (Table 7).

The Northwest Pacific region located in the mid-latitude region and the two groups of data nearly have the same numbers in climate mode, which shows that their CTHs change with the season. The CTH reaches its maximum of around 7.6 km in summer and decreases to a minimum of approximately 5.0 km in winter, followed by spring and autumn. Based on the mean numbers of the two datasets, their maximum relative error is 3% and the others do not exceed 0.4%, which indicate that model 7 possesses strong applicability and credibility.

## 5. Discussion and Conclusions

MODIS is a passive remote sensing sensor which provides a CTH record more than two decades long. CTH is an essential climate variable that impacts the Earth’s energy budget and hydrological cycle. In order to obtain more accurate CTHs from the MODIS data, this study conducted research on remote sensing retrieval using neural network methods based on CALIOP data. Therefore, seven neural network methods were proposed and compared to improve the accuracy of MODIS CTHs.

A comprehensive analysis and evaluation were conducted on seven models belonging to three categories, using multiple indicator parameters. The results from different indicator parameters exhibit slight variations. In terms of the category of input parameters, the third category of models performs best, followed by the first category, while the second category of models has poorer applicability. Specifically, models directly utilizing MODIS product parameters and those combining direct parameters and radiance as input perform better, whereas models relying solely on satellite radiance for inversion yield less satisfactory results. Among the input parameters, cloud top temperature and cloud top pressure are identified as the most critical factors influencing the accuracy of the neural network model retrieval. This also explains the suboptimal performance of the second category of models. Cloud top pressure was usually produced through the CO_2_ slicing method and involved the height information through the Global Data Assimilation System (GDAS) of the National Center for Environmental Prediction (NECP) [75]. Therefore, some cloud product key parameters such as cloud top temperature and cloud top pressure can help improve the accuracy of CTH retrieval from MODIS.

Furthermore, an interesting phenomenon is that when constructing the input neural network model, more input parameters do not necessarily make the model better. However, the results of the model certainly depend on crucial parameters, such as cloud top temperature and cloud top pressure. Model 3 has 3 input parameters, model 1 has 15 parameter inputs, and both models yield comparable retrieved CTHs. Conversely, model 4, with the highest number of input channels (22 radiance inputs), exhibits poorer retrieved results. This may be related to the neural network architecture, and the choice of the numbers of hidden layers and neurons in each layer can significantly affect the performance of the network. Further research needs to be developed. Additionally, the accuracy of CTH retrieval also varies for different height clouds. Overall, model 7 in the third category is best, with eight input parameters, which makes it a recommended choice in CTH retrieval from MODIS data.

Additionally, the neural network model is essentially an empirical model that requires a substantial amount of data for training. In addition to evaluating the model based on statistical indicators, its generalization ability also needs to be tested in practical applications. Therefore, these models are applied to retrieve individual cases during the Typhoon Lekima period in 2019 and around the globe in 2009. All models except models 4 and 5 clearly showed the main structure of the typhoon, including the typhoon eye, eye wall, and spiral arm. However, the performance of each model in the details is different. Among them, the result of model 7 is closest to the real cloud top structure of the typhoon. In the global CTHs, the results retrieved by model 7 with the lowest RMSE are compared with the CTH from CALIPSO data, and they have the same distribution patterns, which means that the neural network method had good applicability and credibility in CTH retrieval. In models 4 and 5, only using MODIS radiance data, a direct relationship was established between satellite observation data and cloud top height, and this reduces the intermediate steps. However, the results were the worst among the models, which is worth further study.

## Figures and Tables

**Table 1:** MODIS spectral channel parameters and primary uses.

Band	Bandwidth (μm)	Spectral Radiance (W/m^2^/μm/sr)	Ground Resolution (m)	Primary Purpose
1	0.620–0.670	21.8	250	Land/Cloud/Aerosol boundaries
2	0.841–0.876	24.7	250	
3	0.459–0.479	35.3	500	Land/Cloud/Aerosol properties
4	0.545–0.565	29.0	500	
5	1.230–1.250	5.4	500	
6	1.628–1.652	7.3	500	
7	2.105–2.155	1.0	500	
8–16	0.405–0.877	-	-	Ocean color/Phytoplankton/ Biogeochemistry
17	0.890–0.920	10.0	1000	Atmospheric water vapor
18	0.931–0.941	3.6	1000	
19	0.915–0.965	15.0	1000	
20	3.660–3.840	0.45 (300 K)	1000	Surface/Cloud temperature
21	3.929–3.989	2.38 (335 K)	1000	
22	3.929–3.989	0.67 (300 K)	1000	
23	4.020–4.080	0.79 (300 K)	1000	
24	4.433–4.498	0.17 (250 K)	1000	Atmospheric temperature
25	4.482–4.549	0.59 (275 K)	1000	
26	1.360–1.390	6.00	1000	Cirrus cloud water vapor
27	6.535–6.895	1.16 (240 K)	1000	
28	7.175–7.475	2.18 (250 K)	1000	
29	8.400–8.700	9.58 (300 K)	1000	Cloud properties
30	9.580–9.880	3.69 (250 K)	1000	Ozone
31	10.780–11.280	9.55 (300 K)	1000	Surface/Cloud temperature
32	11.770–12.270	8.94 (300 K)	1000	
33	13.185–13.485	4.52 (260 K)	1000	Cloud top altitude
34	13.485–13.785	3.76 (250 K)	1000	
35	13.785–14.085	3.11 (240 K)	1000	
36	14.085–14.385	2.08 (220 K)	1000	

**Table 2:** Description of variables from MODIS used to train the neural networks.

Parameter	Variable Names	Unit	Source
Radiance (From reflective solar band-scaled integers)	Radiance 1–7/17–19/26	W/m^2^/μm/sr	MAC02^(^*^1)^
Radiance (From emissive band-scaled integers)	Radiance 20/27–36	W/m^2^/μm/sr	
Cloud phase infrared	CPI	----	MAC06^(^*^2)^
Cloud top pressure	CTP	hPa	
Cloud top temperature	CTT	K	
Cloud top height	CTH	km	
Surface temperature	SFT	K	
Cloud emissivity (From 11/12/13.3/8.5 μm bands)	CE11, CE12, CE13, CE85	----	
Cloud effective radius (Used 2.1/3.7 μm bands)	CER, CER37	m	
Cloud optical thickness (Used 2.1/3.7 μm bands)	COT, COT37	----	
Low-cloud temperature (From IR Window retrieval)	ICT	K	
Cloud water path (Used 2.1/3.7 μm bands)	CWP, CWP37	g/m^2^	

^(*1)^ MAC02 contains the calibrated ground observation data. ^(*2)^ MAC06 is the cloud product data.

**Table 3:** Cloud type matching between MODIS and CALIPSO data.

Code of Cloud Type	Cloud Type Interpretation of CALIPSO	Matched with MODIS Type	Cloud Genera
0	low overcast, transparent	Stratus	Low
1	low overcast, opaque	Stratus	Low
2	transition stratocumulus	Stratocumulus	Low
3	low, broken cumulus	Cumulus	Low
4	altocumulus (transparent)	Altocumulus	Middle
5	altostratus (opaque)	Altostratus/Nimbostratus	Middle
6	cirrus (transparent)	Cirrus/Cirrostratus	High
7	deep convective (opaque)	Deep convection	High

**Table 4:** Summary of datasets used in this study.

Dataset	Count	Type	Mode	Period
Training dataset	11,727,474	Orbit	Daytime	2008
Testing dataset	2,931,869	Orbit	Daytime	2008
Validation dataset	13,339,183	Orbit	Daytime	2009
Single-image dataset	2,748,620	Grid	Daytime	9 August 2019

**Table 5:** Establishment of the neural network models based on input parameters.

Name	Network-Specific Variables	Applicability
Model 1	CTP,CTT,CPI,SFT,CE11,CE12,CE13,CE85,CER,CER37,COT,COT37,ICT,CWP,CWP37	Only Day
Model 2	CTP,CTT,CE11,CE12,CE13,CE85,CPI,SFT	All Time
Model 3	CTP,COT,COT37	Only Day
Model 4	radiance 1–7,17–19,20,26–36	Only Day
Model 5	radiance 4,6,7,20,27,28,29,31,36	Only Day
Model 6	CTP,CTT,radiance 4,6,7,20,27,28,29,31,36	Only Day
Model 7	CTP,CTT,radiance 20,28,31,32,33,36	All Time

**Table 6:** Statistical measures for the RMSE for all models.

Method	Type Ⅰ			Type Ⅱ		Type Ⅲ	
Model	Model 1	Model 2	Model 3	Model 4	Model 5	Model 6	Model 7
Pirmse (%)	24.8	15.3	13.7	−1.7	−29.4	26.4	27.3
Mean Pirmse (%)	17.9			−15.6		26.9	

**Table 7:** Comparison of the two kinds of CTHs in Northwest Pacific region.

	MAM	JJA	SON	DJF
CALIOP (km)	6.66	7.70	6.32	4.89
Model 7 (km)	6.68	7.67	6.35	5.04
Relative error (%)	0.30	0.4	0.3	3.0

## Data Availability

The MODIS data and CALIPSO data were downloaded from the Earth Science Data Systems (ESDS) Program and the internet URL is https://search.earthdata.nasa.gov/search (accessed on 23 January 2020).
